# Fracture of the Spine and Sternum

**Published:** 1842-12-03

**Authors:** 


### ANALECTA.

*Fracture of the Spine and Sternum.*—Three cases of fractured spine, the sternum suffering by contre-coup, are recorded in the annual surgical report of the Hotel-Dieu, published in the " Gazette Médicale." In each of the three cases there was complete separation of the first bone of the sternum from the second, the latter having passed up in front of the former, and causing a considerable projection. The organs of respiration and circulation were unaffected. These cases of fracture, with the exception of those recorded by David and Chaussier, are unique. A painter, while engaged at work on the walls of a church, fell from a height of twenty-five feet, the body in supination and across, on to a bench ; there resulted fracture of the fourth dorsal vertebra, with complete paralysis of sensation and motion, and such a state of tension at either end of the sternum, as to separate completely the first bone from the second, this last bone being thrust forwards by the ribs, which were also propelled and drawn upwards by the contraction of the pectoralis major. The poor fellow survived forty days. Although the fracture was fully reduced, there was not any attempt at union. In a second case, the man, who had fallen from a great height, was brought dead to the hospital. In addition to the separation and displacement of the first and second bones of the sternum, there was complete diastasis of the articulations of the pelvis, considerable ecchymosis of the sub-peritoneal cellular tissue, fracture of the seventh cervical vertebra, and dislocation backwards of the right wrist, with fracture of the carpus. There was not any contusion on the anterior paries of the chest, and from the great separation of the bones of the pelvis, without the lower extemities having been bruised, it may be inferred that the man fell first on the nates, and then on the upper part of the spine, for both sides of the seventh cervical arch were broken. The fracture of the sternum must have resulted from its great tension and the contrecoup.

The third case occurred in the person of a man who fell upon the back, the fifth cervical vertebra being fractured, whence resulted loss of sensation and motion in the upper and lower extremities. The first bone of the sternum was in this case also separated from the second by contre-coup. A singular symptom, that of constant priapism, continued during the five days that the man survived the accident. A fourth part is reported from the Hôpital Cochin, in which the patient, an athletic carrier, fell from a considerable height on his feet on to a scaffold, and then fell back on the spine and head. The result was a grinding of both ossa calcis to powder, fracture of the spine, and of the left side of the cranium, with depression, and simple oblique fracture of the third bone of the sternum. The patient died in two days.—*Prov. Med. Journ., from Gazette des Hôpitaux*, July, 1842.

